# Case Report: Successful treatment of external beam radiation-induced optic papillopathy with intravitreal anti-VEGF

**DOI:** 10.3389/fopht.2023.1144241

**Published:** 2023-04-28

**Authors:** Andrew R. Carey

**Affiliations:** Wilmer Eye Institute, Johns Hopkins University School of Medicine, Baltimore, MD, United States

**Keywords:** radiation, optic neuropathy, optic papillopathy, anti-VEGF (vascular endothelial growth factor), intravitreal (IVT) drug, intravitreal bevacizumab (IVB; Avastin), bevacizumab (Avastin), optic disk edema

## Abstract

Three cases of optic disc edema arising from radiation optic neuropathy isolated to the intra-ocular optic nerve following external beam radiation for head and neck squamous cell carcinoma are presented. A literature review of the etiology, presentation, and treatment is included for discussion, along with proposed diagnostic criteria.

## Introduction

Radiation toxicity to the optic nerve is a known and feared complication of therapeutic radiation indicated for head and neck tumors as well as orbital and CNS tumors (1). Radiation-induced optic neuropathy is more commonly encountered with CNS radiation, with a predilection for the intracranial segment of the optic nerve (1). However, toxicity can affect the intraocular segment of the optic nerve, which is most commonly associated with radiation therapy for intraocular tumors. Various terms have been used for radiation optic neuropathy involving the intraocular optic nerve. It is often termed simply radiation optic neuropathy, for example when discussing the complication of choroidal melanoma, it is often referred to simply as radiation optic neuropathy, but it has also been referred to as anterior radiation optic neuropathy. However, this term is not specific enough to rule out retrobulbar extension, and, therefore, the term radiation-induced optic papillopathy (RIOP) is used in this manuscript. There is limited information in the literature about the visual outcomes and treatment of RIOP associated with the treatment of non-ocular tumors. This paper reports three cases to provide further information on presentation and treatment.

## Case series

### Case #1

**Figure 1:**
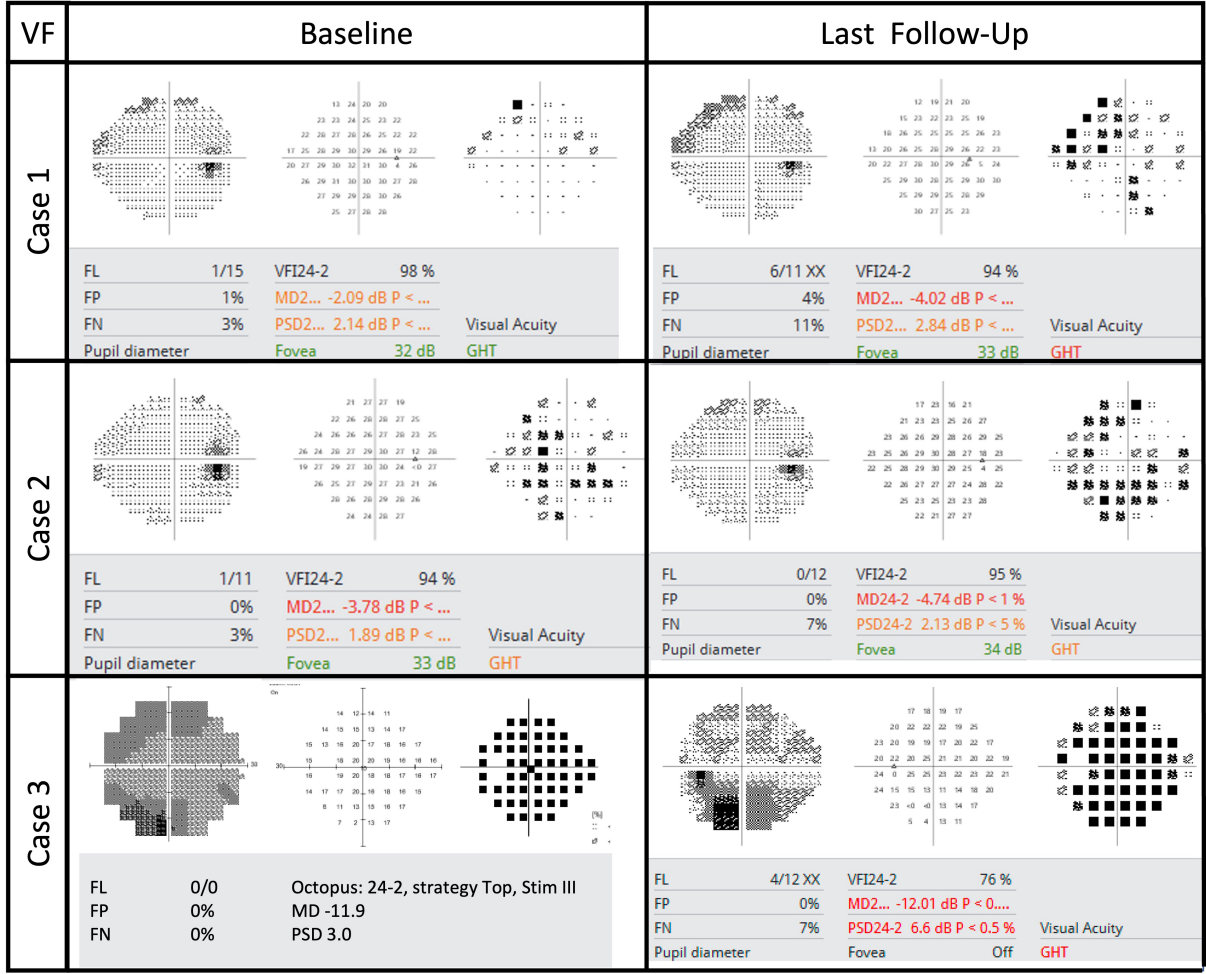
Automated visual fields at baseline and final follow-up.

**Figure 2:**
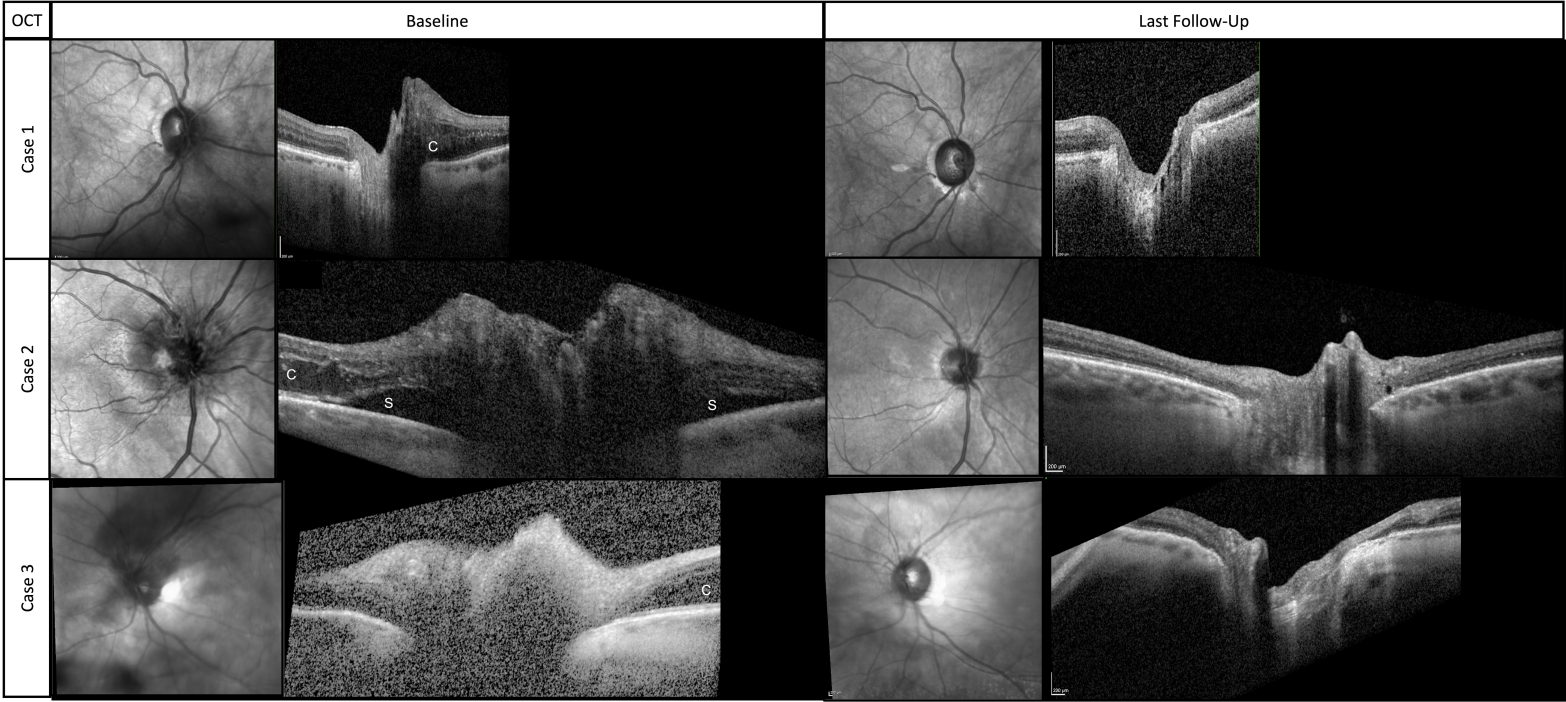
Near-infrared images and associated cross-sectional OCT scans through the optic disc at baseline and final follow-up. (c) indicates cystoid changes from intraretinal fluid. (s) indicates subretinal fluid.

**Figure 3:**
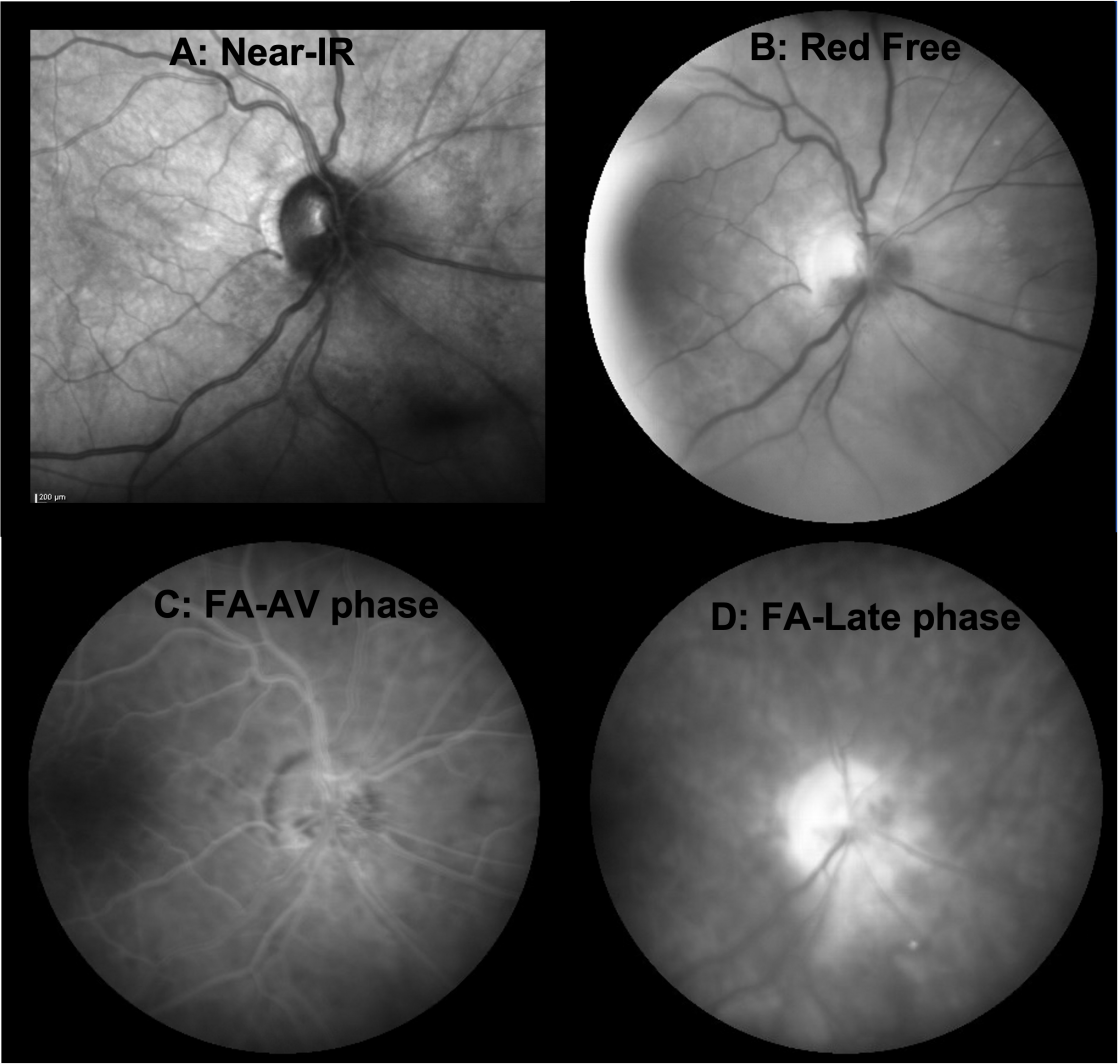
*En face* imaging of the acute phase of RIOP from case 1. **(A)** Near-infrared image acquired during OCT scanning of the optic nerve showing clustered pinpoint hyporeflectivity surrounding the optic disc from 12 to 8 o’clock, corresponding to cystoid changes on cross-sectional OCT but no frank optic disc edema. **(B)** Red-free photograph highlighting the hemorrhage within the optic disc from 2 to 8 o’clock. **(C)** Fluorescein angiogram in the arteriovenous phase, which shows hypofluorescence due to blockage from the hemorrhage within the optic disc along with telangiectatic vessels on the optic disc but no neovascularization. **(D)** Fluorescein angiogram in the late stage demonstrating diffuse optic disc hyperfluorescence from staining, some inferonasal leakage, and some residual blocking from hemorrhage.

A woman in her early 80s presented with a 1-month history of blurred vision in the right eye. Thirty-nine months prior to her initial presentation she was treated for cutaneous squamous cell carcinoma (SqCCa) of the periorbita, which was deemed unresectable, and was treated with a total of 72 Gy of intensity-modulated (fractionated) external beam radiation (IMRT). Examination of the right eye showed visual acuity of 20/30, color vision of 12/14 Ishihara plates, relative afferent pupillary defect (RAPD) of < 0.3 log-units, and normal ocular motility and alignment. Fundus examination of the right eye showed a red hemorrhage within the optic disc from 3 to 7 o’clock, spilling over the disc rim, and which was associated with mild inferior disc edema, and a remote retinal blot hemorrhage at 6 o’clock. The left eye was normal. Automated visual field (AVF) testing showed faint superior arcuate scotoma (**Figure 1**); an optical coherence tomography (OCT) cube scan, centered on the optic disc, demonstrated nasal and inferior thickening with cystoid changes and intraretinal hyperreflective foci (**Figure 2**). Red-free photos highlighted the hemorrhage within the optic disc; fluorescein angiography showed blockage from the hemorrhage and telangiectatic vessels on the optic disc, with diffuse disc staining and profuse fluorescein dye leakage from the optic disc nasally and inferiorly (**Figure 3**). MRI of the orbits with and without contrast showed possible trace optic disc elevation but no retrobulbar enhancement.

The patient was diagnosed with RIOP and was treated with intravitreal bevacizumab (IVB). The optic disc edema resolved after four doses of monthly IVB. The patient was treated with a treat-and-extend protocol for a total of eight doses and followed up for 13 months after her last injection. At the last follow-up, visual acuity was 20/25, AVF testing showed a superior arcuate defect with a relatively stable mean deviation of –4 dB with a baseline value of –2.1 dB, and OCT showed that the thickness of the peripapillary retinal nerve fiber layer (RNFL) had improved from the baseline value of 118 to 86 μm (**Figure 2**).

### Case #2

A woman in her early 60s presented with a 5-month history of blurred vision in the right eye. Fifty months prior, she had completed IMRT for SqCCa of the right lacrimal sac, receiving a total dose of 72 Gy, combined with chemotherapy. Her SqCCa was complicated by metastases to the right parotid gland and cervical lymph nodes, which were treated with 67 Gy. Examination of the right eye demonstrated visual acuity of 20/25, color vision of 12/14 Ishihara plates, no detectable RAPD with a 0.3 log-unit neutral density filter, and normal ocular motility and alignment. Fundus examination of the right eye revealed 360 degrees of optic disc edema, worst superiorly, with telangiectatic blood vessels, a tiny cotton wool spot, a few nerve fiber layer hemorrhages, and exudates and thickening in the nasal macula. AVF testing revealed an enlarged blind spot and general depression with a mean deviation of –3.8 dB, and OCT demonstrated 360 degrees of peripapillary subretinal fluid and nasal cystoid macular edema (**Figure 2**). MRI of the orbits with and without contrast was negative for optic nerve enhancement.

The patient was diagnosed with RIOP and was treated with IVB. The optic disc edema resolved after six doses of monthly IVB. The patient was treated with a treat-and-extend protocol for a total of nine doses and followed up for 12 months after her last injection. At the last follow-up, visual acuity was 20/25, AVF testing showed a superior arcuate defect with a relatively stable mean deviation of –4.7 dB with a baseline level of –3.8 dB (**Figure 1**), and OCT showed that the thickness of the RNFL had improved from 246 μm at baseline to 75 μm (**Figure 2**).

### Case #3

A woman in her early 60s presented with a 1-month of blurred vision in the left eye with new floaters. Thirty months prior, she had completed IMRT combined with chemotherapy for perineural invasion by cutaneous SqCCa involving the left side of her face and the trigeminal nerve for a total dose of 72 Gy. SqCCa had initially been diagnosed 5 years prior, at which time she underwent Mohs surgery; perineural invasion was initially detected 3 years prior and was being treated with cemiplimab (anti-programmed cell death receptor 1 monoclonal antibody). Examination of the left eye demonstrated visual acuity of 20/100; color vision of 14/14 Ishihara plates; 0.3 log-unit RAPD; ptosis and 25%–50% limitation of elevation, adduction, and depression; telangiectatic skin changes over the brow and upper eyelid; 360 degrees of mild corneal neovascularization with guttae and central haze; and mild cataracts. Dilated fundus examination revealed a hazy view, mild vitreous hemorrhage extending from the optic disc, and hemorrhage within the nerve fiber layer extending from the optic disc. Ancillary testing included AVF testing, which showed generalized depression with a mild inferior scotoma on pattern deviation and a mean deviation of –11.9 dB (**Figure 1**); an OCT cube scan centered on the optic disc was notable for superonasal cystoid changes extending into the macula and mild diffuse thickening of the peripapillary RNFL (**Figure 2**). MRI of the skull base protocol with and without contrast demonstrated no retrobulbar optic nerve infiltration.

The patient was diagnosed with RIOP and was treated with IVB. The optic disc edema resolved after four doses of monthly IVB. The patient was treated with a treat-and-extend protocol for a total of seven doses, and the last follow-up was 30 months after her last injection. At the final follow-up, visual acuity was 20/60, AVF testing showed general depression and mild inferior scotoma, the mean deviation was stable at –12 dB, with a baseline of –11.9 dB (**Figure 1**), and the OCT RNFL thickness measurement improved from 118 μm at baseline to 76 μm (**Figure 2**).

**Table 1** provides details of all three cases. Ages are reported in decade form to protect the patients’ privacy.

**Table 1:** Radiation-induced optic papillopathy case series.

	Case 1	Case 2	Case 3
Cancer diagnosis	Cutaneous facial SqCCa	Lacrimal sac SqCCa	Cutaneous facial SqCCa with perineural invasion
Total radiation dose	72 Gy	72 Gy	72 Gy
Decade of age at diagnosis of optic neuropathy (years)	80s	60s	60s
Time from radiation to optic neuropathy diagnosis (months)	39	50	30
Vasculopathic risk factors	Hypertension	Hypertension. dyslipidemia	None
Other ocular conditions	PCIOL, OAGS, DBN, DCR	PCIOL, dry eyes	Cataract, 3NP, recurrent HSV keratitis, exposure keratopathy
Visual acuity at diagnosis	20/30	20/25	20/100
Color vision at diagnosis	12/14	12/14	14/14
Visual field at diagnosis (dB)	MD –2.1; mild superior arcuate	MD –3.8; enlarged blind spot, general depression	MD –12; general depression
Optic disc appearance at diagnosis	Inferior edema	Diffuse edema	Superior edema, overlying VH
OCT RNFL thickness at diagnosis (microns)	118, infranasal cystoid changes	246, peripapillary subretinal fluid with cystoid changes extending into macula	118, cystoid changes extending into macula
Treatment course	TAE, eight doses of IVB	TAE, nine doses of IVB	TAE, seven doses of IVB
Time to optic disc edema resolution post first injection (months)	4	6	5
Duration of follow-up after last anti-VEGF (months)	13	12	30
Visual acuity at last follow-up	20/25	20/25	20/60
Visual field at last follow-up (dB)	MD –4; superior arcuate	MD –4.7; faint inferior arcuate	MD –12.1; general depression
OCT RNFL thickness at last follow-up (μm)	86, normal	75, superonasal thinning	76, superior broad thinning, inferior focal thinning

SqCCa, cutaneous squamous cell carcinoma; PCIOL, posterior chamber intraocular lens; OAGS, open-angle glaucoma suspect; DBN, downbeat nystagmus; DCR, dacryocystorhinostomy; 3NP, third nerve palsy; VH, vitreous hemorrhage; HSV, herpes simplex virus; OCT, optical coherence tomography; RNFL, retinal nerve fiber layer; MD, mean deviation; TAE, treat and extend; IVB, intravitreal bevacizumab; VEGF, vascular endothelial growth factor.

## Discussion

Little is published about the treatment of radiation-induced optic neuropathy limited to the intraocular segment of the optic nerve (optic papillopathy, RIOP). The most common clinical scenario of RIOP encountered is following brachytherapy for choroidal melanoma, partly due to the frequency of choroidal melanoma, the high-dose exposure in proximity to the optic nerve, and the fact that patients getting frequent eye exams while monitoring for tumor progression and complications such as radiation retinopathy. Head and neck SqCCa, particularly if it involves periorbital structures such as cutaneous lesions and those originating in the sinuses and nasopharynx, as seen in all three cases reported in this series, is also particularly high risk due to the high total dose required for local tumor control in combination with proximity to the optic nerves, resulting in a high maximum point dose to the optic nerves (1).

Traditionally, RIOP associated with brachytherapy has been treated by retinal specialists using methods similar to radiation retinopathy with intravitreal anti-VEGF and intravitreal steroids, but without evidence from randomized controlled trials (RCTs) (2). Eckstein et al., in 2019, called into question the long-term benefits of intravitreal injections in the setting of proton therapy for choroidal melanoma; however, these patients are treated with a high dose per fraction (14–15 Gy equivalent), which is much higher than the standard dose per fraction. This results in increased toxicity to both the macula and the optic nerve, compared with treatment for head and neck and CNS cancers, which may account for the poorer visual prognosis (1, 3). Systemic (intravenous as well as intra-arterial) bevacizumab has been used to treat CNS radiation toxicity, including in an RCT of both saline placebo and steroids and a case series in patients who have failed steroids, anticoagulation, and hyperbaric oxygen (4–7). Systemic bevacizumab for the treatment of retrobulbar radiation-induced optic neuropathy has been studied in multiple case series, but none has included an RCT (1). Intravitreal treatment for RIOP was selected in these case series due to the ability to deliver the medication to the site of injury and limit systemic exposure and toxicities, as compared with systemic administration.

The optimal treatment protocol for RIOP is also not clear. In the case of radiation retinopathy, some authors recommend continuous treatment for radiation retinopathy on the basis that, although presenting manifestations will resolve with treatment, late findings of vasculopathy including microaneurysms and capillary non-perfusion can still develop (8). Other retinal vascular diseases, such as diabetes and retinal vein occlusions, can be treated successfully with an as-needed regimen, starting with monthly intravitreal injections and, once intraretinal and subretinal fluid have resolved, pausing treatments, resuming only in the event of vision-threatening recurrence of exudation (9). A third option, known as treat and extend, similarly begins with monthly injections, but once fluid has resolved the treatments are spread out, at 2-week intervals (unless exudation recurs, at which point the interval is shortened), typically for 12–14 weeks, at which time treatment can be continued for maintenance or paused and the patient monitored for recurrence (10). The as-needed treatment may result in fewer treatments; however, if exudation recurs, the interval between treatments could be longer, and, therefore, risk more severe exudation, which may cause an irreversible decline in visual acuity. Treat and extend was recommended for the patients reported in this series, as recurrent optic disc edema may result in compartment syndrome and permanent optic nerve damage and vision loss; in addition, it was felt that RIOP is likely an ischemic disease and a longer treatment course may help allow disease control during the vascular remodeling process.

Short-term results of intravitreal steroids for RIOP have been described by the Shields group from nine patients secondary to plaque brachytherapy for choroidal melanoma (11). The mean time from radiation to RIOP was 18 months, ranging from 6 to 33 months. Eight patients were treated with a single injection and one patient received two injections 6 months apart. The optic disc edema resolved at a mean of 4 months after injection (ranging from 1 to 11 months). Shields et al. reported that 78% of patients had improved or stabilized vision (five out of nine improved).

Results of IVB for RIOP have been described by Finger and Chin from 14 patients secondary to plaque brachytherapy for choroidal melanoma (12). The mean time from radiation to RIOP was 42 months, ranging from 11 to 114 months. The mean number of injections was 11, ranging from 2 to 21, given every 6–8 weeks, over a mean period of 22 months. The majority (11/14) of patients received continuous treatment. Treatment was stopped only if the patient died or was non-adherent, or if treatment was futile (no improvement from baseline in ability to count fingers). Optic discs were stabilized in a mean of 5 months. Vision was stabilized or improved in 64% of patients (7/14 improved).

Roelofs et al. investigated a combination of bevacizumab and triamcinolone for RIOP in nine patients secondary to plaque brachytherapy for choroidal melanoma (13). The mean time from radiation to RIOP was 17 months, ranging from 8 to 28 months. The mean time to the resolution of the disc edema was 7 months. The authors divided their patients into those with acute vision loss (*n* = 4) and those without (*n* = 5). Improvement was experienced by 100% of the acute vision loss group, and 80% of those without vision loss showed stabilization (although in two of these cases, stabilization was at visual acuity of 20/400 and hands motion at baseline).

There has been a single case report of the use of intravitreal bevacizumab for RIOP following external beam radiation therapy for head and neck cancer (14). The patient developed vision loss 6 years after radiation, with visual acuity reduced to 20/50 with a combination of RIOP and maculopathy with subretinal fluid. Following treatment with a single intravitreal injection of bevacizumab, visual acuity improved to 20/20 within 2 weeks.

Clear diagnostic criteria do not exist in the literature. Proposed diagnostic criteria based on the literature and the cases within this report are as follows: (1) optic disc edema in the affected eye with hemorrhages involving the nerve fiber tissue within the margin of the optic disc; (2) evidence of optic nerve dysfunction (i.e., reduced visual acuity or color vision, or nerve fiber bundle-type visual field defect); (3) no signs of retrobulbar optic nerve involvement on MRI (no enhancement or diffusion-weighted imaging signal changes); (4) no signs of tumor infiltration or compression; (5) completion of radiation treatment at least 6 months prior with involvement of the optic disc in the field of radiation; and (6) no alternative cause, such as retinal vascular occlusion, infectious optic papillitis/neuroretinitis, optic nerve infiltration/compression, or inflammatory optic neuritis.

Radiation retinopathy may be used in conjunction with RIOP. It is important to distinguish RIOP from retrobulbar optic nerve disease because intravitreal treatment is unlikely to provide the necessary therapeutic doses to the retrobulbar optic nerve, which could result in undertreatment and progressive optic neuropathy with progressive vision loss. Therefore, MRI of the orbits with and without contrast with fat suppression is recommended.

RIOP may be difficult to distinguish from non-arteritic anterior ischemic optic neuropathy (NAION), and, in truth, there is probably a non-vasculitis ischemic component to RIOP. The two features that best distinguish NAION from RIOP are (1) the atypical hemorrhages within the optic disc and (2) the peripapillary exudation, both of which are out of proportion to the extent of the optic disc edema and were demonstrated in all three of these cases. Persistent optic disc edema may be seen past the typical time expected in NAION, as demonstrated in case 2. This patient had optic disc edema 5 months after symptom onset. All patients had optic disc edema for an additional 3–5 months once treatment was initiated (the patient in case 2 had a total duration of optic disc edema of 10 months). In addition, the visual field defects seen in these three cases are not typical for NAION, and the RAPDs were all very small. It is possible that patients with RIOP may present with more severe vision loss that may more closely mimic NAION or even arteritic disease. Oncology patients are often immunosuppressed, and, therefore, at increased risk of atypical infections; with newer checkpoint inhibitors they may develop atypical optic neuritis, which should be included in the differential diagnosis (15, 16).

The exact etiology of RIOP, or any manifestation of radiation-induced optic neuropathy, is not fully understood. Among the proposed etiologies are direct neural injury, including apoptosis induction, oligodendrocyte injury, and vascular injury with a combination of ischemia and vascular incompetence leading to leakage (1, 7, 17). A vascular etiology is particularly supported when relying on radiation retinopathy as a model for neuropathy in which capillary non-perfusion, reduced retinal blood flow, increased oxygen saturation, and vascular leakage can be demonstrated *in vivo*. (18, 19)

Certainly, there are limitations to this study, including the small numbers and lack of a control group. There are no historical controls or natural history studies of patients with RIOP due to treatment of non-ocular tumors with which to compare our findings. A larger series with longer-term follow-up from multiple institutions would be of benefit, and, in an ideal world, an RCT comparing placebo and intravitreal steroids would be of greatest benefit; however, given the rarity of this condition, this would prove quite challenging. In addition, studies are needed to better elucidate risk factors and ideal surveillance regimens.

In conclusion, RIOP is a rare complication of therapeutic radiation for the head and neck as well as CNS tumors marked by atypical optic disc edema and hemorrhage with peripapillary exudation without retrobulbar involvement. Successful treatment with IVB is possible, and early recognition is key, as the final visual outcome likely depends on vision at the time of treatment initiation. Optimal treatment protocols have not been developed, and further research is required, likely necessitating multicenter studies owing to the rarity of the disease.

## Data availability statement

The original contributions presented in the study are included in the article/supplementary material. Further inquiries can be directed to the corresponding author.

## Ethics statement

Ethics review and approval were not required for the study on human participants in accordance with the local legislation and institutional requirements. Written informed consent for participation was not required for this study in accordance with the national legislation and the institutional requirements.

## Author contributions

The author confirms being the sole contributor of this work and has approved it for publication.
